# Sleep complaints in early pregnancy. A cross-sectional study among women attending prenatal care in general practice

**DOI:** 10.1186/s12884-020-2813-6

**Published:** 2020-02-22

**Authors:** Ruth K. Ertmann, Dagny R. Nicolaisdottir, Jakob Kragstrup, Volkert Siersma, Melissa C. Lutterodt

**Affiliations:** Research Unit for General Practice and Section for General Practice, Institute of Public Health, University of Copenhagen, Øster Farimagsgade 5, DK-1014 Copenhagen K, Denmark

**Keywords:** Pregnancy, Sleep, Primary health care, Pregnancy-related symptoms, General practice, Nausea, Back pain, Pelvic girdle pain, Pelvic cavity pain

## Abstract

**Background:**

Sleep problems in late pregnancy are common, but sleep in early pregnancy is less well described. The aim of this study was to describe the occurrence and severity of sleep complaints in early pregnancy. We asked the women about worries due to sleep problems. Furthermore, we investigated the associations between sleep complaints and pregnancy-related symptoms. This association was studied taking into account physical and mental health, sociodemographic characteristics, and reproductive history of the women.

**Methods:**

Cross-sectional study in Danish general practice based on an electronic questionnaire completed by pregnant women and a Pregnancy Health Record filled in by the general practitioner (GP). The questionnaire measured three sleep complaints and 11 common physical pregnancy-related symptoms. The sleep complaints were measured as mild, moderate or severe, and it was recorded how much they worried the women. The associations between the physical pregnancy-related symptoms and sleep complaints were assessed by odds ratios from multivariable logistic regression models.

**Results:**

The questionnaire was completed by 1338 out of 1508 eligible women before the end of gestation week 16. The gestational age ranged from 5 to 16 weeks (median 11 weeks) among the included women. On average, more than one third of the women reported to have at least one of the three sleep complaints in the questionnaire. Problems “taking a long time to fall asleep” was reported by 312 women (23%), “waking up too early” was reported by 629 (47%), and 183 (14%) had been “lying awake most of the night”. One sleep complaint was reported by 38%, two by 16, and 4% had all three symptoms. The majority were not at all or only mildly worried because of their sleep disturbances, but moderate or severe worries were found among 46% of those“ taking a long time to fall asleep” and among 40% of those “lying awake most of the night”. “Moderate or severe complaints” were reported by 277 (21%) women “Moderate or severe complaints” were associated with pregnancy-related physical symptoms, such as back pain, pelvic girdle pain and pelvic cavity pain, but only the association with pelvic cavity pain stayed significant after adjustment for depression.

**Conclusion:**

This study showed that sleep complaints in early pregnancy are common, and sleep complaints showed association with physical as well as mental symptoms. It may be important for pregnant patients that clinicians address depression, and mood in relation to sleep problems during pregnancy.

## Background

In many countries, care for pregnant women is shared between general practitioners (GPs), midwives, and obstetric hospital departments. The roles may vary, but often the GP has an important function as coordinator of care, and for many women the GP is the sole provider of health care in the early stage of pregnancy. These early consultations are an opportunity to prepare the woman for the rest of the pregnancy, screen for problems and to discuss her concerns. However, knowledge about the experience of women’s health concerns in early pregnancy, such as nausea, vomiting, itching, back pain and sleep complaints is often based on personal clinical experience and relatively small clinical studies.

Sleep disturbances in late pregnancy have been described in several studies [1] and may be explained by profound physiological and anatomical changes such as foetal movements, musculoskeletal discomfort and nycturia [2]. Only a few have studied sleep complaints in early pregnancy, but disturbances may be prevalent [1, 3]. In early pregnancy, sleep complaints may be caused by physical symptoms such as nausea, vomiting or pain. Such physical feelings may be simple explanations for change in sleep patterns, but complaints may have a more complex background that includes hormonal, mechanical, emotional and sociological factors [4–6]. A review studying short sleep duration, poor sleep quality and insomnia during pregnancy found that these conditions related to a number of adverse pregnancy outcomes, such as prenatal depression, gestational diabetes, pre-eclampsia, abnormal length of labour, cesarean delivery, alteration in fetal growth and preterm birth. The mechanisms behind are unclear, but sleep disturbances may produce changes in the hypothalamic-pituitary-adrenal axis and abnormal immune/inflammatory reactions [7]. Further, sleep is critical for glucose metabolism and several meta-analyses found that both self-reported and objectively measured sleep is assoiated with hyperglycemia and an increased risk of gestational diabetes mellitus (GDM) [8–10]. Sleep is also important for blood pressure; both short and long sleep duration as well as poor sleep quality are associated with elevated blood pressure [11], measured with Pittsburgh Sleep Quality Index (PSQI) [12]. Lastly, a systematic review found evidence for the impact of poor sleep on mental health [13]. Up to 85% of pregnant women want to discuss pregnancy-related symptoms during the preventive health checks performed by their general practitioner (GP) or the midwife [14]. It may, therefore, be important for GPs and midwives to address sleep problems early in pregnancy. To do this, knowledge about sleep problems in pregnant women is needed, and it is important to be aware of any worries sleep problems may produce.

The aim of this study was to describe the occurrence and severity of sleep complaints in early pregnancy (≤ gestation week 16). We asked the women about worries due to sleep problems. Furthermore, we investigated the associations between sleep complaints and pregnancy-related symptoms. This association was studied, taking into account the physical and mental health, sociodemographic characteristics, and reproductive history of the women.

## Methods

### Study design

Cross-sectional study comprising questionnaires and Pregnancy Health Records for pregnant women participating in the first prenatal care visit to their GPs.

### Setting

The healthcare system in Denmark is tax-funded, and care is free of charge for the patient. The majority of Danes (99%) are registered with a GP who functions as gatekeeper to secondary care. A minimum of three prenatal care visits and one postpartum are offered by the GP. The first consultation is offered at gestational age 6–10 weeks to all women who wish to go through with their pregnancy. This consultation, which precedes other contacts to the healthcare system, is accepted by almost 100% of women. In this consultation a thorough and structured record is established (the Pregnancy Health Record) and sent to midwives and hospital departments. The second and third prenatal care visits usually take place at pregnancy weeks 25 and 32, and the postpartum examination 8 weeks after birth.

### Sampling of the pregnant women

GPs were recruited from two of the five Danish administrative Regions (The Capital Region of Denmark and Region Zealand) with a total of 1561 practices, organised in 53 geographical units. Among these, 19 units were randomly selected. The 308 practices situated in the selected geographical area were individually contacted and asked to participate in the study. A total of 192 practices agreed to participate. The practices could be either single-handed or partnerships. Among the 190 participating practices, 117 were active in the study and recruited one or more pregnant women to the study. The 117 active practices comprised a total of 294 GPs.

All pregnant women booking an appointment for the first prenatal care visit with one of the participating GPs were eligible for inclusion in the project. The women received oral and written information on the project and were consecutively included after signing a consent form. The inclusion period was from 1 April 2015 to 15 August 2016. Women were excluded if they did not complete the electronic questionnaire (all in Danish), if they withdrew consent or if the pregnancy ended in abortion.

### Data collection

Data were collected from the Pregnancy Health Record and from an electronic patient questionnaire. The electronic questionnaire was sent to the women after the first prenatal care visit. The questionnaire could only be returned when it was fully completed. Nonrespondents were sent two reminders and some were contacted by phone.

#### Pregnancy health record

The Pregnancy Health Record was filled in by the GP at the first prenatal care visit. The Pregnancy Health Record is a standardised two-page record introduced by the health authorities in Denmark [15]. In this study, the following parts of the record are used: lifestyle habits, reproductive background and previous psychiatric disorders. A copy of the record to be used in the project was received from the GPs.

#### Questionnaire

Only questionnaires completed before or in gestational week 16 were used for this study. The questionnaire measured three sleep symptoms: 1) Has it taken you a long time to fall asleep in the past week? (Categorised as: no, mild, moderate, severe). 2) Have you been waking up too early in the past week? (no, mild, moderate, severe). 3) Have you been lying awake most of the night in the past week? (no, mild, moderate, severe). In relation to all three sleep symptoms, the women were further asked whether they had been worried about the symptoms (no, mildly, moderately, severely). The sleep questions used in our study derive from the Nottingham Health Profile (NHP) [16]. Moreover, the questions have been used and showed good reliability and validity in two Danish Breast Cancer studies [17, 18].

Furthermore, the questionnaire asked about a selection of pregnancy-related physical symptoms including nausea, vomiting, back pain, pelvic girdle pain, pelvic cavity pain, vulvar itching, varicose veins, leg cramps, pregnancy itching, vaginal bleeding and uterine contractions; answers were categorised as present or not present. The questionnaire contained anatomical pictures with arrows pointing at e.g. point of pelvic girdle pain.

Other information obtained from the questionnaire and the Pregnancy Health Record was used to adjust for confounding and arranged in three blocks**:** Block I - sociodemographics: marital status (married, cohabiting, single), children living at home (no, yes), education (primary, secondary and higher education), occupation (employed, student, other, unemployed, sick leave), income of household (< 39,999 EUR, 40000–79,999 EUR, 80000–119,999 EUR, ≥120,000 EUR, do not want to answer) and age (5-years grouping). Lifestyle habits: smoking during pregnancy (no, yes), drinking alcohol during pregnancy (no, yes) and use of drugs (no, yes).

Block II - physical health: self-assessed health (very good, good, fair, poor, very poor), self-assessed fitness (very good, good, fair, poor, very poor). The block with health-related data also included information from the Pregnancy Health Record concerning reproductive background: parity (0, 1,> 1), previous abortions (0, 1,> 1), in vitro fertilisation (no, yes) and week of gestation.

Block III - mental health: previous psychological difficulties (no, yes - but no health care, yes - with health care), known psychiatric disease (no, yes), and symptoms of depression and anxiety. Depression was measured with the Major Depression Inventory, (MDI) [19]. The MDI contains 10 items measured on a 6-point scale from 0 (never) to 5 (all the time) with in a time frame of the past 2 weeks. The total score of the MDI has a theoretical range from 0 (no depression) to 50 (extreme depression). A total score of 21 or more is indicative of a mild depressive episode according to ICD-10 [19]. Cronbach’s alpha of the MDI is 0.89 [20]. Anxiety was measured with the Anxiety Symptom Scale (ASS), which is constructed analogue to the MDI, covering 10 items for measuring the states of anxiety [19]. The total ASS score has a theoretical range from 0 (no anxiety) to 50 (extreme anxiety).

### Statistical analyses

Characteristics of the study population were compared between women without sleep complaints and those with sleep complaints. Multivariable logistic regression analysis was used to test the associations between “moderate or severe sleep complaints” and pregnancy-related physical symptoms. Women were labelled as having “moderate or severe sleep problems”, if they had reported a moderate or severe problem in response to one or more of the three sleep questions. The associations were assessed in five ways: unadjusted, adjusted for each of the three blocks individually, and adjusted for all three blocks simultaneously. The purpose of these adjustments was to see whether the additional information could explain the associations apparent in the unadjusted assessment. Only women with complete records and questionnaires were included in the regression analysis.

A *p*-value < 0.001 was considered statistically significant. We have specifically lowered the level of significance from the usual 0.05 to 0.001 to minimise the risk of finding spurious associations because of multiple testing. The statistical analyses were performed with SAS version 9.4 (SAS Institute, Cary, NC, USA).

## Results

A total of 1508 pregnant women gave informed consent to participate in the study. We received completed questionnaires from 1455 (96%), Pregnancy Health Records for 1479 women (98%), and both datasets were received for 1442 women (96%). Women who answered the first questionnaire after week 16 were excluded, leaving 1338 women for the analysis. The gestational age of the women at the time of completing the questionnaire was between 5 and 16 weeks (range) and the median was week 11.

The demographic characteristics and health of participating women are described in Table 1.

**Table 1:** Participant demographics

	Women with moderate or severe sleep complaints		Women without sleep complaints		χ^2^ -Test	
	(*n* = 277)	%	(*n* = 1061)	%	Test statistic	*p*-value
Questionnaire						
Married or cohabiting	260	93.8	1030	97 .0	6.798	0.0334
Children living at home	153	55.2	626	59.0	1.281	0.2739
Education level:						
Primary education	90	32.5	319	30.1	12.263	0.0065
vSecondary education	74	26.7	347	32.7		
Higher education	42	15.2	207	19.5		
Occupation:						
Employed	187	67.5	812	76.5	14.530	0.0058
Student	55	19.9	142	13.4		
Other	13	4.7	41	3.9		
Unemployed	13	4.7	53	5.0		
Sick leave	9	3.2	13	1.2		
Household income:						
< 39,999 EUR	46	16.6	119	11.2	12.681	0.0129
40,000-79,999	90	32.5	316	29.8		
80,000-119,999	80	28.9	365	34.4		
> 120,000	19	6.9	121	11.4		
Do not want to answer	42	15.2	140	13.2		
Self-rated health:						
Very good	23	8.3	172	16.2	68.090	<.0001
Good	149	53.8	719	67.8		
Fair	92	33.2	154	14.5		
Poor	13	4.7	16	1.5		
Very poor	0	0.0	0	0.0		
Self-assessed physical form / fitness:						
Very good	4	1.4	39	3.7	20.712	0.0004
Good	60	21.7	283	26.7		
Fair	123	44.4	504	47.5		
Poor	78	28.2	220	20.7		
Very poor	12	4.3	15	1.4		
History of psychological difficulties:						
Yes but I did not seek treatment	60	21.7	204	19.2	28.773	<.0001
Yes, and I did seek treatment	113	40.8	279	26.3		
Depression	113	40.8	112	10.6	143.577	<.0001
Anxiety	47	17.0	48	4.5	51.563	<.0001
Pregnancy Health Record						
Age:						
< 25 years	48	17.3	123	11.6	9.883	0.0196
26–30 years	101	36.5	363	34.2		
31–35 years	76	27.4	373	35.2		
36+ years	52	18.8	202	19.0		
Smoking during pregnancy	18	6.5	72	6.8	0.029	1.000
Alcohol during pregnancy	2	0.7	7	0.7	0.013	1.000
Use of recreational drugs	1	0.4	2	0.2	0.292	0.5017
Psychiatric disease	32	11.6	65	6.1	9.618	0.0038
Fertility treatment	35	12.6	95	9.0	3.394	0.0689
Abortion:						
Miscarriage, one spontaneous	43	19.5	125	11.8	6.570	0.1604
Miscarriage, several times	23	8.3	78	7.4		
Induced abortion	53	19,2	171	16.1		
Given birth:						
Yes, once	102	36.8	390	36.8	2.080	0.3535
Yes, several times	41	14.8	194	18.3		
Gestation age, weeks:						
5–10 weeks	109	40.4	435	42.1	0.2790	0.5974
11–16 weeks	161	59.6	597	57.9		

Characteristics of all 1338 participating women and the 277 women with significant sleep problems (one or more” moderate or severe sleep complaints”)

Footnote: We have missing gestation age data from 36 women. 29 of these women did not have a sleep problem and 7 women did have one. These were included in the analysis because with a high likelihood they have a gestation age less than 17 weeks

Women with sleep complaints had worse self-rated health and worse self-rated fitness. Furthermore, they had experiences of previous psychological difficulties, higher depression and anxiety scores.

On average, more than one third of the pregnant women reported to have at least one of the three sleep complaints in the questionnaire. A single sleep complaint was reported by 509 (38%), two by 219 (16%), and 59 (4%) had all three complaints. Three hundred and twelve women (23%) had to some degree “taking a long time to fall asleep”, 629 (47%) had been “waking up too early” and 183 (14%) had been “lying awake most of the night” (Table 2). The majority were not or only mildly worried because of their sleep disturbances, but moderate or severe worries were found among 46% of those “taking a long time to fall asleep” and among 40% of those “lying awake most of the night” (Table 2).

**Table 2:** Sleep questions

	Does not have the sleep problem		Does have the sleep problems							
			No complaints		Mild complaints		Moderate complaints		Severe complaints	
	n	(%)	n	(%)	n	(%)	n	(%)	n	(%)
“Fall asleep”										
Has it taken you a long time to fall asleep in the past week?	1026/1338	77%	30/312	9%	171/312	55%	68/312	22%	43/312	14%
Have you been worried* about taking along time to fall asleep?			0/30	0%	1/171	1%	11/68	16%	13/43	30%
“Waking up too early”										
Have you been waking up too early in the past week?	709/1338	53%	124/629	20%	334/629	53%	116/629	18%	55/629	9%
Have you been worried* about waking up too early?			0/124	0%	1/334	0%	16/116	14%	5/55	9%
“Lying awake most of the night”										
Have you been lying awake most of the night in the past week?	1155/1338	86%	5/183	3%	58/183	32%	55/183	30%	65/183	35%
Have you been worried* about lying awake most of the night?			0/5	0%	1/58	2%	8/55	15%	16/65	25%

Answers to questions about sleep problems (*n* = 1338). The answers were divided into two groups: no problems (“does not have the sleep problem”) or when problems were present the women were asked to grade the problem as no, mild, moderate or severe complaints. The percentage shown for the graded problems are subgroups of the whole group concerning problems, while the numbers are exact. Those with a problem were also asked if the problem had worried them (“no, mildly, moderately or severely”)

“Moderate or severe sleep complaints” were reported by 277 women (21%). These women were younger, had shorter education, lower household income, lower self-rated health, lower self-rated fitness, and they had more psychological and psychiatric problems (Table 1).

The primary unadjusted analysis showed statistically significant (*P* < 0.001) associations between “moderate or severe sleep complaints” and the following pregnancy-related physical symptoms: back pain, pelvic girdle pain, pelvic cavity pain (Table 3). No significant associations with sleep complaints were found for nausea, vomiting, vaginal bleeding, itching, varicose veins, or uterine contractions. Table 3 shows the results of the logistic regression analyses, which is adjusted for other characteristics of the 1338 women. The associations between pregnancy-related symptoms and sleep complaints all remained statistically significant, when age and sociodemographic characteristics of the woman were included in the analysis (Block I) and when reproductive background and current pregnancy were included in the analysis (Block III). However, when adjusted for physical and mental health (Block II), none of the associations remained statistically significant. We analysed the reason for this weakening of the associations by adjusting these associations for the variables that were related to sleep complaints as well as to the pregnancy-related symptoms in Block II individually: previous psychological difficulties, depression score, anxiety score, and self-assessed fitness. Depression score appeared to explain the weakening of the associations to the greatest extent.

**Table 3:** The associations between “moderate or severe sleep complaints”, other pregnancy-related symptoms and other characteristics of the pregnant women

	N(%)	N(%) with significant sleep complaints	Unadjusted	Block I: Adjusted for age and sociodemographic characteristics	Block II: Adjusted for physical and mental health status and resources	Block III: Adjusted for reproductive background	Adjusted for all blocks
Variable			**OR [95%CI]**	**OR [95%CI]**	**OR [95%CI]**	**OR [95%CI]**	**OR [95%CI]**
Nausea	1182 (88.34%)	259 (93.50%)	2.15 [1.29–3.58]	2.07 [1.23–3.49]	1.57 [0.92–2.69]	2.19 [1.31–3.65]	1.56 [0.90–2.72]
Pelvic cavity pain	755 (56.43%)	189 (68.23%)	1.88 [1.42–2.49]*	1.82 [1.36–2.43]*	1.65 [1.22–2.24]	1.87 [1.41–2.50]*	1.61 [1.17–2.21]
Vomiting	527 (39.39%)	117 (42.24%)	1.16 [0.89–1.52]	1.10 [0.83–1.45]	0.89 [0.66–1.20]	1.14 [0.87–1.49]	0.83 [0.61–1.14]
Back pain	483 (36.10%)	132 (47.65%)	1.84 [1.41–2.41]*	1.75 [1.32–2.31]*	1.39 [1.03–1.87]	1.83 [1.39–2.39]*	1.36 [1.00–1.84]
Pelvic girdle pain	438 (32.74%)	119 (42.96%)	1.75 [1.34–2.30]*	1.69 [1.28–2.24]*	1.42 [1.06–1.92]	1.75 [1.33–2.30]*	1.41 [1.03–1.92]
Vulvar itching	240 (17.94%)	56 (20.22%)	1.21 [0.87–1.69]	1.18 [0.84–1.66]	1.02 [0.70–1.47]	1.23 [0.88–1.72]	1.02 [0.70–1.49]
Vaginal bleeding	230 (17.19%)	60 (21.66%)	1.45 [1.04–2.01]	1.44 [1.03–2.02]	1.44 [1.01–2.07]	1.45 [1.04–2.02]	1.43 [0.99–2.08]
Pregnancy itching	210 (15.70%)	44 (15.88%)	1.02 [0.71–1.46]	0.92 [0.63–1.33]	0.91 [0.61–1.35]	1.00 [0.69–1.44]	0.82 [0.55–1.24]
Leg cramp	112 (8.37%)	34 (12.27%)	1.76 [1.15–2.70]	1.63 [1.04–2.55]	1.36 [0.84–2.18]	1.77 [1.15–2.72]	1.32 [0.80–2.17]
Uterine contractions	41 (3.06%)	11 (3.97%)	1.42 [0.70–2.87]	1.60 [0.77–3.32]	1.42 [0.65–3.10]	1.55 [0.76–3.20]	1.68 [0.74–3.80]
Varicose veins	31 (2.32%)	7 (2.53%)	1.12 [0.48–2.63]	1.30 [0.54–3.09]	0.92 [0.36–2.35]	1.26 [0.53–2.99]	1.15 [0.43–3.05]

The associations between sleep complaints and a number of other pregnancy-related symptoms among the 277 women who reported one or more “moderate or severe sleep complants”. For each pregnancy-related symptom the results of five logistic regression analyses are reported: unadjusted, adjusted separately for age and sociodemographic characteristics (Block I), adjusted for physical and mental health status and resources (Block II), adjusted for reproductive background (Block III), and adjusted for all three blocks simultaneously. Associations with a p-value < 0.001 were considered statistically significant and marked with *

## Discussion

### Statement of principal findings

On average, more than one third of pregnant women experienced some degree of poor sleep in early pregnancy, although most symptoms were rated as minor. Waking up too early was reported by 47% of the women, but few were worried. On the other hand, 46% of the women who had been “taking a long time to fall asleep”, and 40% of the women who had been “lying awake most of the night” were worried to a moderate or severe degree. Sleep complaints showed association with other pregnancy-related symptoms, such as back pain, pelvic girdle pain and pelvic cavity pain, but only the association with pelvic cavity pain stayed significant after adjustment for depression.

### Limitations of the study

No exclusion criteria were used, but with regard to representativity for the general population of women in early pregnancy a number of factors must be considered. The questionnaire was in Danish and results may be less representative for non-native women. The GPs participating in the study were sampled by a systematic procedure based on random selection from two representative regions of Denmark, including urban and rural areas, and areas with low as well as high social status. This strengthens the geographical representation of the study, but the GP practices that voluntarily chose to contribute constitute only approximately 40% of those asked. There were very few nonrespondents among women who accepted to participate, and complete data were obtained from almost all participants. However, not all eligible women may have been asked, and not all women managed to have had their first prenatal care visit between pregnancy weeks 6–10, where the inclusion was supposed to take place. This delay in receiving the signed consent form with the women’s e-mail from the GPs led to a delay in answering among the participating women. Those women were asked to recall how they experienced symptoms and feelings around week 9–10.

We assessed sleep complaints by means of questions to the pregnant women. Such subjective measures of sleep duration and sleep efficiency have previously been shown to be correlated with objective measures [10, 21], but may obviously be affected by the individual’s reporting style. The degree of trouble and the level of worries can, however, only be assessed by the women themselves. Unfortunately, we do not have pre-conception information about sleep complaints and, therefore, cannot be sure that problems are different post-conception. Significantly more mid-sleep awakenings have, however, previously been found in pregnant women compared to non-pregnant women [22].

The most commonly used postpartum instrument is the Edinburgh Postnatal Depression Scale (EPDS), which was developed to measure postpartum depression. For our study we used the MDI because it is more often used in clinical practice by GPs in Denmark. MDI has been demonstrated to have good validity and reliability, and only includes is a single question about sleep [23]. Cronbach’s Alpha value for the MDI in our study was 0.84 and for the ASS was 0.80. Factors associated with sleep problems were measured with validated scales when possible (e.g. sociodemographic status (https://www.dst.dk/da/).

### Findings in context of existing research

Problems with sleep in late pregnancy have been described in many studies and may affect more than 2/3 of women [24, 25]. The enlarged uterus and movements of the child may, for example, explain this. Studies from early pregnancy are fewer and the population size of our study is relatively large. We found poor sleep quality among 47% of the women. This is lower compared to 74% poor sleepers among 346 self-selected women in the first trimester in a recent web-based study [26], and other small cross-sectional studies have found frequencies between 28 and 38% [27] and 60% [28]. Sleep complaints in the non-pregnant populations seem to be less frequent than among first trimester pregnant women. A national sleep survey of Australian adults reported that 33–45% had inadequate sleep [29], and a Japanese study found the frequencies to be around 29% [30].

We found associations between sleep complaints and some pregnancy-related physical symptoms, leading us to explore possible mechanisms through which sleep in pregnancy may impact health or vice versa. A study exploring mid-sleep awakenings found no associations with nausea and indigestion [22]. Similarly, we found no association with nausea. Another study found that women experiencing distressing physical symptoms during pregnancy more often suffer from poorer sleep quality and more symptoms of depression [31], which corresponds well with our result showing association with sleep complaints and mood. Furthermore, our women had higher depression score (MDI score > 20) 8 weeks postpartum, if they experienced physical discomfort in early pregnancy, such as back pain and pelvic cavity pain [32]. Pain as well as depression affect sleep, and depression may interact with pregnant women’s subjective impression of pregnancy-related pain and their sleep experiences. This could be described as maternal stress, inasmuch as several studies have found that sleep problems relate to maternal stress. These heterogeneity studies show associations with elevated blood pressure [11], childhood abuse [33], intimate partner violence [34] and higher levels of symptoms of anxiety and depression, both during pregnancy and postpartum [13, 35]. However, it is debatable how strong this relation is, as it seems that our women were not so distressed that the sleep complaint worried them. Nevertheless, some of the pregnant women may have been worried, because those who experienced poor sleep quality had a high use of health care [36]. Overall, GPs have a unique opportunity for reducing maternal distress by introducing sleep intervention, which represents a potential low-cost, non-pharmacological prevention and treatment strategy for postpartum mental illness, by articulating that the pregnant women should be aware of the connection between sleep and symptoms of depression and anxiety [13, 37, 38]. In addition, the women should be encouraged to engage in physical activity and exercise during pregnancy, as studies have shown that this has an overall beneficial effect on sleep characteristics and sleep continuity and on the woman’s mental state [39, 40].

## Conclusion

This study showed that sleep complaints in early pregnancy are common, and that they did not seem to worry the women a great deal. Sleep complaints also showed association with physical as well as mental symptoms.

### Implications of the findings

Sleep complaints in early pregnancy are common and should be addressed by the GP and midwife at the first prenatal care visit. Anxiety and mood may be related and relevant topics for discussion in relation to sleep problems.

## Supplementary information

**Additional file 1.** Questionnaire.

## Data Availability

The datasets used and/or analysed during the present study are available from the corresponding author on request.

## Abbreviations

Ass: Anxiety Symptom Scale
GPs: General practitioners
MDI: Major Depression Inventory
