# Supplementary material for: Sleep complaints in early pregnancy. A cross-sectional study among women attending prenatal care in general practice
Source: BMC Pregnancy Childbirth. 2020 Feb 22;20:123. doi: 10.1186/s12884-020-2813-6 (PMC7036174; doi:10.1186/s12884-020-2813-6)
Supplement: Supplementary file 1 — Additional file 1. Questionnaire. [file 12884_2020_2813_MOESM1_ESM.docx]

English translation of the reduced questionnaire to the manuscript “Sleep complaints in early pregnancy”. A cross-sectional study among women attending prenatal care in general practice.

| **Welcome to the study!**  **We are pleased that you would like to participate in the survey:**  **“Pregnancy, discomfort, and mental well-being during pregnancy and in the period after giving birth”.**  Velkommen til undersøgelsen!  Vi er glade for, at du vil deltage i undersøgelsen:  "Graviditet- Gener og psykisk velbefindende under graviditeten og i perioden efter  fødslen". |
| --- |

| **The following questions are regarding pregnancy-related physical symptoms.** De følgende spørgsmål handler om almindelige graviditetsgener. |
| --- |

Have you been feeling nauseous so far in your pregnancy? Har du indtil videre i din graviditet, haft kvalme?

(1) ❑ No, not at all (Nej, slet ikke)

(2) ❑ Yes, mildly (Ja, lidt)

(3) ❑ Yes, moderately (Ja, noget)

(4) ❑ Yes, severely (Ja, meget)

**if problem:** Have you been worried about the nausea? **Har du været bekymret over, at du har haft kvalme?**

(1) ❑ No, not at all (Nej, slet ikke)

(2) ❑ Yes, mildly (Ja, lidt)

(3) ❑ Yes, moderately (Ja, noget)

(4) ❑ Yes, severely (Ja, meget)

Have you been vomiting so far in your pregnancy? Har du indtil videre i din graviditet, haft opkastninger?

(1) ❑ No, not at all (Nej, slet ikke)

(2) ❑ Yes, mildly (Ja, lidt)

(3) ❑ Yes, moderately (Ja, noget)

(4) ❑ Yes, severely (Ja, meget)

if problem: Have you been worried about the vomiting? Har du været bekymret over, at du har kastet op?

(1) ❑ No, not at all (Nej, slet ikke)

(2) ❑ Yes, mildly (Ja, lidt)

(3) ❑ Yes, moderately (Ja, noget)

(4) ❑ Yes, severely (Ja, meget)


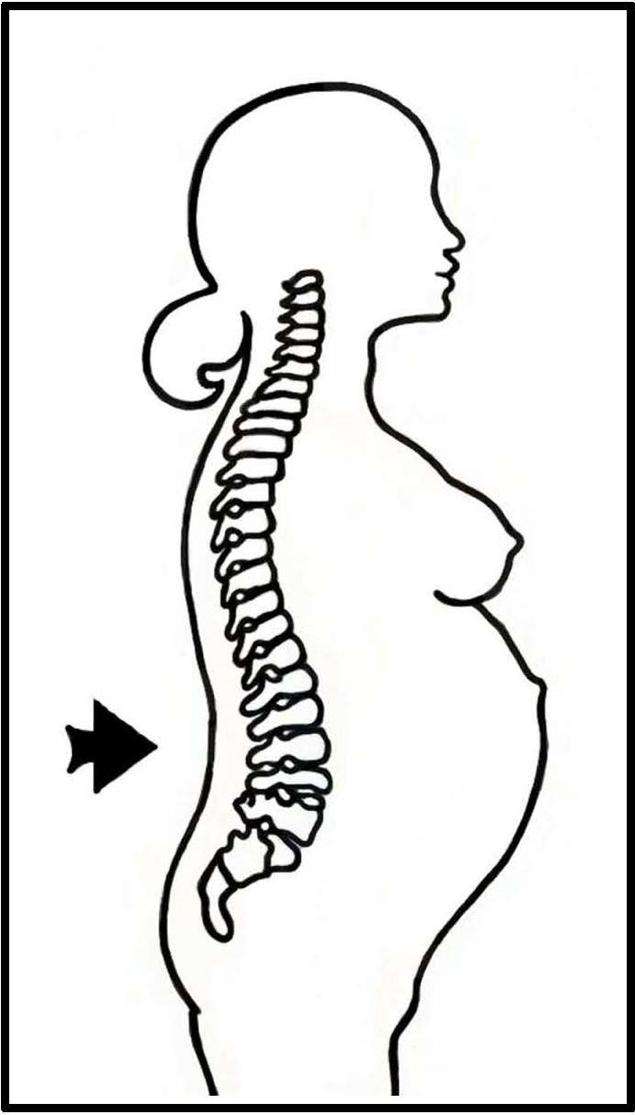


Have you had back pain so far in your pregnancy? Har du indtil videre i din graviditet, haft rygsmerter?

(1) ❑ No, not at all (Nej, slet ikke)

(2) ❑ Yes, mildly (Ja, lidt)

(3) ❑ Yes, moderately (Ja, noget)

(4) ❑ Yes, severely (Ja, meget)

if problem: Have you been worried about the back pain? Har du været bekymret over, at du har haft rygsmerter?

(1) ❑ No, not at all (Nej, slet ikke)

(2) ❑ Yes, mildly (Ja, lidt)

(3) ❑ Yes, moderately (Ja, noget)

(4) ❑ Yes, severely (Ja, meget)


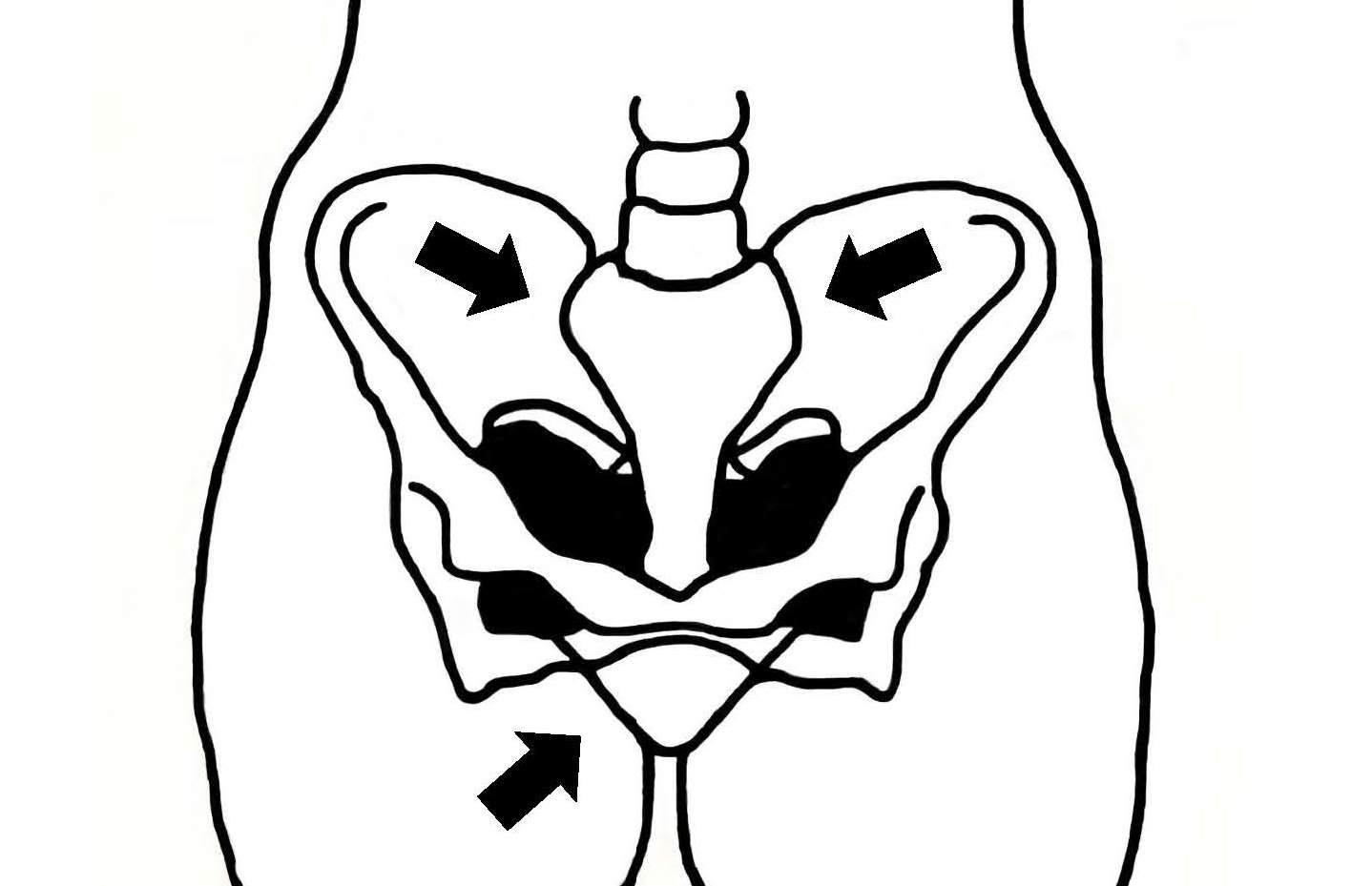


Have you had pelvic pain so far in your pregnancy? Har du indtil videre i din graviditet, haft smerter i bækkenet?

(1) ❑ No, not at all (Nej, slet ikke)

(2) ❑ Yes, mildly (Ja, lidt)

(3) ❑ Yes, moderately (Ja, noget)

(4) ❑ Yes, severely (Ja, meget)

if problem: Have you been worried about your pelvic pain? Har du været bekymret over, at du har haft bækkensmerter?

(1) ❑ No, not at all (Nej, slet ikke)

(2) ❑ Yes, mildly (Ja, lidt)

(3) ❑ Yes, moderately (Ja, noget)

(4) ❑ Yes, severely (Ja, meget)

Have you had lower abdominal pain so far in your pregnancy? Har du indtil videre i din graviditet, haft smerter i underlivet?

(1) ❑ No, not at all (Nej, slet ikke)

(2) ❑ Yes, mildly (Ja, lidt)

(3) ❑ Yes, moderately (Ja, noget)

(4) ❑ Yes, severely (Ja, meget)

**if problem:** Have you been worried about the lower abdominal pain? **Har du været bekymret over, at du har haft smerter i underlivet?**

(1) ❑ No, not at all (Nej, slet ikke)

(2) ❑ Yes, mildly (Ja, lidt)

(3) ❑ Yes, moderately (Ja, noget)

(4) ❑ Yes, severely (Ja, meget)

Have you had vaginal bleeding so far in your pregnancy? Har du indtil videre i din graviditet, blødt fra skeden?

(1) ❑ Yes (Ja)

(2) ❑ No (Nej)

**if problem:** In which gestational week have you been bleeding? **I hvilken graviditetsuge(r) har du blødt?**

________________________________________
________________________________________
________________________________________
________________________________________
________________________________________
________________________________________

**if problem:** Have you been worried about the bleeding? **Har du været bekymret over, at du har blødt?**

(1) ❑ No, not at all (Nej, slet ikke)

(2) ❑ Yes, mildly (Ja, lidt)

(3) ❑ Yes, moderately (Ja, noget)

(4) ❑ Yes, severely (Ja, meget)

if problem: **Have you had any pain associated with the bleeding?** Har du haft smerter i forbindelse med blødningen ?

(1) ❑ No, not at all (Nej, slet ikke)

(2) ❑ Yes, mildly (Ja, lidt)

(3) ❑ Yes, moderately (Ja, noget)

(4) ❑ Yes, severely (Ja, meget)

if problem: Have you been worried about the pain? Har du været bekymret over, at du har haft smerter?

(1) ❑ No, not at all (Nej, slet ikke)

(2) ❑ Yes, mildly (Ja, lidt)

(3) ❑ Yes, moderately (Ja, noget)

(4) ❑ Yes, severely (Ja, meget)

Have you had vaginal itching so far in your pregnancy? Har du indtil videre i din graviditet, haft kløe i skridtet?

(1) ❑ No, not at all (Nej, slet ikke)

(2) ❑ Yes, mildly (Ja, lidt)

(3) ❑ Yes, moderately (Ja, noget)

(4) ❑ Yes, severely (Ja, meget)

**if problem:** Have you been worried about the itching? **Har du været bekymret over, at du har haft kløe i skridtet?**

(1) ❑ No, not at all (Nej, slet ikke)

(2) ❑ Yes, mildly (Ja, lidt)

(3) ❑ Yes, moderately (Ja, noget)

(4) ❑ Yes, severely (Ja, meget)

Have you experienced getting varicose veins so far in your pregnancy? Har du indtil videre i din graviditet, haft åreknuder?

(1) ❑ No, not at all (Nej, slet ikke)

(2) ❑ Yes, mildly (Ja, lidt)

(3) ❑ Yes, moderately (Ja, noget)

(4) ❑ Yes, severely (Ja, meget)

if problem: Have you been worried about the varicose veins? Har du været bekymret over, at du har fået åreknuder?

(1) ❑ No, not at all (Nej, slet ikke)

(2) ❑ Yes, mildly (Ja, lidt)

(3) ❑ Yes, moderately (Ja, noget)

(4) ❑ Yes, severely (Ja, meget)

Have you had pregnancy itching so far in your pregnancy? Har du indtil videre i din graviditet, oplevet graviditetskløe?

(1) ❑ No, not at all (Nej, slet ikke)

(2) ❑ Yes, mildly (Ja, lidt)

(3) ❑ Yes, moderately (Ja, noget)

(4) ❑ Yes, severely (Ja, meget)

**if problem:** Have you been worried about the pregnancy itching? **Har du været bekymret over, at du har haft graviditetskløe?**

(1) ❑ No, not at all (Nej, slet ikke)

(2) ❑ Yes, mildly (Ja, lidt)

(3) ❑ Yes, moderately (Ja, noget)

(4) ❑ Yes, severely (Ja, meget)

Have you had leg cramps so far in your pregnancy? **Har du indtil videre i din graviditet, haft kramper i læggene?**

(1) ❑ No, not at all (Nej, slet ikke)

(2) ❑ Yes, mildly (Ja, lidt)

(3) ❑ Yes, moderately (Ja, noget)

(4) ❑ Yes, severely (Ja, meget)

**if problem:** Have you been worried about the leg cramps? **Har du været bekymret over, at du har haft lægkramper?**

(1) ❑ No, not at all (Nej, slet ikke)

(2) ❑ Yes, mildly (Ja, lidt)

(3) ❑ Yes, moderately (Ja, noget)

(4) ❑ Yes, severely (Ja, meget)

Have you had uterine contractions so far in your pregnancy? **Har du indtil videre i din graviditet, haft plukveer?**

(1) ❑ No, not at all (Nej, slet ikke)

(2) ❑ Yes, mildly (Ja, lidt)

(3) ❑ Yes, moderately (Ja, noget)

(4) ❑ Yes, severely (Ja, meget)

**if problem:** Have the uterine contractions been painful? **Har plukveerne gjort ondt?**

(1) ❑ Yes (Ja)

(2) ❑ No (Nej)

**(If problem):** Have you been worried about the uterine contractions? **Har du været bekymret over, at du har haft smertefulde plukveer?**

(1) ❑ No, not at all (Nej, slet ikke)

(2) ❑ Yes, mildly (Ja, lidt)

(3) ❑ Yes, moderately (Ja, noget)

(4) ❑ Yes, severely (Ja, meget)

| **In the following, we have some questions about how you sleep at night. Please keep in mind that the questions concern the past week!** |
| --- |

Has it taken you a long time to fall asleep in the past week? Har du den sidste uges tid været længe om at falde i søvn?

(1) ❑ No, not at all (Nej, slet ikke)

(2) ❑ Yes, mildly (Ja, lidt)

(3) ❑ Yes, moderately (Ja, noget)

(4) ❑ Yes, severely (Ja, meget)

**(if problem):** Have you been worried, that it took you long time to fall asleep? **Har du været bekymret over, at du har været længe om at falde i søvn?**

(1) ❑ No, not at all (Nej, slet ikke)

(2) ❑ Yes, mildly (Ja, lidt)

(3) ❑ Yes, moderately (Ja, noget)

(4) ❑ Yes, severely (Ja, meget)

**Have you been waking up too early in the morning in the past week?** Har du den sidste uges tid vågnet alt for tidligt om morgenen?

(1) ❑ No, not at all (Nej, slet ikke)

(2) ❑ Yes, mildly (Ja, lidt)

(3) ❑ Yes, moderately (Ja, noget)

(4) ❑ Yes, severely (Ja, meget)

(if problem): Have you been worried, that you woke up too early in the morning? Har du været generet af, at du er vågnet alt for tidligt om morgenen?

(1) ❑ No, not at all (Nej, slet ikke)

(2) ❑ Yes, mildly (Ja, lidt)

(3) ❑ Yes, moderately (Ja, noget)

(4) ❑ Yes, severely (Ja, meget)

Have you been lying awake most of the night in the past week? Har du den sidste uges tid ligget vågen det meste af natten?

(1) ❑ No, not at all (Nej, slet ikke)

(2) ❑ Yes, mildly (Ja, lidt)

(3) ❑ Yes, moderately (Ja, noget)

(4) ❑ Yes, severely (Ja, meget)

**(if problem):** Have you been worried, that you were lying awake most of the night? **Har du været bekymret over, at du har ligget vågen?**

(1) ❑ No, not at all (Nej, slet ikke)

(2) ❑ Yes, mildly (Ja, lidt)

(3) ❑ Yes, moderately (Ja, noget)

(4) ❑ Yes, severely (Ja, meget)

| **In the following, we have some questions about your health and physical shape.** Nu følger nogle spørgsmål om helbred og fysisk form. |
| --- |

How do you evaluate your health? Hvordan synes du, dit helbred er?

(1) ❑ Very good (Virkeligt godt)

(2) ❑ Good (Godt)

(3) ❑ In between (Nogenlunde)

(4) ❑ Poor (Dårligt)

(5) ❑ Very poor (Meget dårligt)

How do you evaluate your physical fitness? Hvordan synes du, din fysiske form (kondition) er?

(1) ❑ Very good (Virkeligt godt)

(2) ❑ Good (Godt)

(3) ❑ In between (Nogenlunde)

(4) ❑ Poor (Dårligt)

(5) ❑ Very poor (Meget dårligt)

Have you ever had mental difficulties? (Anxiety attacks, depressive symptoms, eating disorders, difficulties in relationships, difficulties in working relationships, etc.) Har du nogensinde oplevet at have psykiske vanskeligheder? (fx angstanfald, nedtrykthed, spiseforstyrrelser, vanskeligheder i parforholdet, vanskeligheder i arbejdsrelationer osv.)

(1) ❑ Yes (Ja)

(2) ❑ No (Nej)

**if problem:** What kind of difficulties did you have? **Hvilke vanskeligheder drejede sig om?**

comment: ____

if problem: Did you receive treatment? Modtog du behandling?

(1) ❑ Yes (Ja)

(2) ❑ No (Nej)

**if problem:** From whom did you receive treatment? You may tick off more than one answer **Hos hvem fik du behandling? Sæt gerne flere krydser**

(1) ❑ General practitioner (Praktiserende læge)

(2) ❑ Psychiatrist (Psykiater)

(3) ❑ Psychologist (Psykolog)

(4) ❑ Alternative treatment (Alternativ behandlig)

(5) ❑ Other (Andet)

| **In the following, we have some questions about your family** Nu kommer der nogle spørgsmål om din familie. |
| --- |

Whom do you live with? Hvem bor du sammen med?

|  | Yes | No |
| --- | --- | --- |
| Spouse/ partner (Ægtefælle / partner) | (1) ❑ | (2) ❑ |
| Own children (biological or adopted) (Egne børn (biologiske eller adopterede)) | (1) ❑ | (2) ❑ |
| Children, who are not mine (partners’ children, foster children, adopted children) (Børn, som ikke er mine egne (partners børn/plejebørn/adopterede)) | (1) ❑ | (2) ❑ |
| Other (Andre) | (1) ❑ | (2) ❑ |

|  |
| --- |

| **The following questions concern your socioeconomic status.** Nu drejer spørgsmålene sig om socio-økonomiske forhold. |
| --- |

How many years did you go to school school? (How many years of schooling did you receive?)**Hvor mange år har du gået i skole?**

_____

Are you currently studying? **Er du i gang med en uddannelse?**

(1) ❑ Yes

(2) ❑ No

If currently studying: What education? Hvilken uddannelse?

(1) ❑ Primary education (Erhversfaglig uddannelse eller tilsvarende praktisk uddannelse, 1-3 år)

(2) ❑ Secondary education (Kort videregående boglig uddannelse, 3-4 år)

(3) ❑ Higher education (Lang videregående uddannelse / universitetsuddannelse, 5 år eller længere)

What was your household’s income in Danish kroner (DKK) before tax during last year? Hvad er din husstands samlede inkomst i danske kroner før skat sidste år?

(1) ❑ Less than 39.999 EURO (Under 300.000 kr.)

(2) ❑ Between 40.000-79.999 EURO (Mellem 300.000-599.000kr)

(3) ❑ Between 80-119.999 EURO (599.000-899.999)

(4) ❑ 120.000 EURO or more (Over 899.999)

(5) ❑ Do not want to answer (Ønsker ikke at svare)

What is your occupation? Hvad er din erhvervsmæssige stilling?(Sæt kryds ved det, der bedst beskriver din erhvervsmæssige stilling)

(1) ❑ Employed (Lønmodtager)

(2) ❑ Self-employed (Selvstændig erhvervsdrivende)

(3) ❑ Both employed and self-employed (Både lønmodtager og selvstændig erhvervsdrivende)

(4) ❑ Unemployed (Arbejdsløs)

(5) ❑ Primary school student (Skoleelev)

(6) ❑ Student (Studerende)

(7) ❑ Apprentice (Lærling)

(8) ❑ Assisting spouse (Medhjælpende ægtefælde)

(9) ❑ In job programme (I revalidering)

(10) ❑ Housewife (Hjemmegående husmor)

(11) ❑ Sick leave (3 months or more) (langtidssygemeldt (3 måneder eller mere))

(12) ❑ Other (Andet)

| **The next questions concern your health and your mood.** De næste spørgsmål handler om dit helbred og dit humør. |
| --- |

**How much of the time during the past two weeks have you been bothered by**

|  | All the time | Most of the time | Slightly more than half of the time | Slightly less than half of the time | Some of the time | At no time |
| --- | --- | --- | --- | --- | --- | --- |
| Nervousness, tension or inner unrest | (5) ❑ | (4) ❑ | (3) ❑ | (2) ❑ | (1) ❑ | (0) ❑ |
| Worrying too much about even the most insignificant things in your daily life? | (5) ❑ | (4) ❑ | (3) ❑ | (2) ❑ | (1) ❑ | (0) ❑ |
| Having to avoid certain things, places, or activities as anxiety-provoking? | (5) ❑ | (4) ❑ | (3) ❑ | (2) ❑ | (1) ❑ | (0) ❑ |
| Incipient anxiety attacks (panic)? | (5) ❑ | (4) ❑ | (3) ❑ | (2) ❑ | (1) ❑ | (0) ❑ |
| Actual anxiety attacks? | (5) ❑ | (4) ❑ | (3) ❑ | (2) ❑ | (1) ❑ | (0) ❑ |
| Recurrent, unpleasant compulsive thoughts that won’t stop? | (5) ❑ | (4) ❑ | (3) ❑ | (2) ❑ | (1) ❑ | (0) ❑ |
| Having to check everything you do, again and again? | (5) ❑ | (4) ❑ | (3) ❑ | (2) ❑ | (1) ❑ | (0) ❑ |
| Feeling very shy in company, for example when eating or drinking in front of other people? | (5) ❑ | (4) ❑ | (3) ❑ | (2) ❑ | (1) ❑ | (0) ❑ |
| Recurrent thought or memories of a very furious experience? | (5) ❑ | (4) ❑ | (3) ❑ | (2) ❑ | (1) ❑ | (0) ❑ |
| Difficulty in performing your daily activities because of these symptoms | (5) ❑ | (4) ❑ | (3) ❑ | (2) ❑ | (1) ❑ | (0) ❑ |

How much of the time in the last two weeks..

|  | All the time | Most of the time | Slightly more than half the time | Slightly less than half the time | Some of the time | At no time |
| --- | --- | --- | --- | --- | --- | --- |
| Have you felt low in spirits or sad? | (5) ❑ | (4) ❑ | (3) ❑ | (2) ❑ | (1) ❑ | (0) ❑ |
| Have you lost interest in your daily activities? | (5) ❑ | (4) ❑ | (3) ❑ | (2) ❑ | (1) ❑ | (0) ❑ |
| Have you felt lacking in energy and strength? | (5) ❑ | (4) ❑ | (3) ❑ | (2) ❑ | (1) ❑ | (0) ❑ |
| Have you felt less self-confident? | (5) ❑ | (4) ❑ | (3) ❑ | (2) ❑ | (1) ❑ | (0) ❑ |
| Have you had a bad conscience or feelings of guilt? | (5) ❑ | (4) ❑ | (3) ❑ | (2) ❑ | (1) ❑ | (0) ❑ |
| Have you felt that life wasn’t worth living? | (5) ❑ | (4) ❑ | (3) ❑ | (2) ❑ | (1) ❑ | (0) ❑ |
| Have you had difficulties concentrating, e.g. when reading the newspaper or watching TV? | (5) ❑ | (4) ❑ | (3) ❑ | (2) ❑ | (1) ❑ | (0) ❑ |
| Have you felt very restless? | (5) ❑ | (4) ❑ | (3) ❑ | (2) ❑ | (1) ❑ | (0) ❑ |
| Have you felt subdued or slowed down? | (5) ❑ | (4) ❑ | (3) ❑ | (2) ❑ | (1) ❑ | (0) ❑ |
| Have you been sleeping too little or too much? | (5) ❑ | (4) ❑ | (3) ❑ | (2) ❑ | (1) ❑ | (0) ❑ |
| Have you suffered from reduced appetite? | (5) ❑ | (4) ❑ | (3) ❑ | (2) ❑ | (1) ❑ | (0) ❑ |
| Have you suffered from increased appetite? | (5) ❑ | (4) ❑ | (3) ❑ | (2) ❑ | (1) ❑ | (0) ❑ |

Do you have anything else you would like to add? Har du ellers noget, du har lyst til at tilføje?

_____

| **Thank you very much for your participation.** Mange tak for din deltagelse. |
| --- |
